# Roles of clonal parental effects in regulating interspecific competition between two floating plants

**DOI:** 10.3389/fpls.2022.924001

**Published:** 2022-07-22

**Authors:** Wen-Han Yu, Li-Min Zhang, Fang-Li Luo, Fei-Hai Yu, Mai-He Li

**Affiliations:** ^1^Institute of Wetland Ecology and Clone Ecology/Zhejiang Provincial Key Laboratory of Plant Evolutionary Ecology and Conservation, Taizhou University, Taizhou, China; ^2^School of Ecology and Nature Conservation, Beijing Forestry University, Beijing, China; ^3^Forest Dynamics, Swiss Federal Research Institute WSL, Birmensdorf, Switzerland

**Keywords:** clonal plants, DNA demethylation, epigenetic inheritance, maternal effect, nutrients, transgenerational plasticity

## Abstract

Parental effects can influence offspring fitness, which may further impact interspecific competition. However, few studies have tested the role of clonal parental effects in regulating interspecific interactions and examined the underlying mechanisms. We conducted two consecutive experiments with two clonal plants (*Pistia stratiotes* and *Eichhornia crassipes*). In the first experiment, the mother ramet of *P. stratiotes* and *E. crassipes* were grown in two nutrient levels and treated with a DNA demethylation reagent (5-azacytidine) or not. In the second experiment, the offspring ramets from each of the four treatments in the first experiment were grown alone (no competition) or with a heterospecific neighbor (with interspecific competition). We found no parental nutrient effect on the competitive ability of *E. crassipes*, but a significant parental nutrient effect of both *E. crassipes* and *P. stratiotes* on the competitive ability of *P. stratiotes*. Furthermore, the parental nutrient effect of *P. stratiotes* on the competitive ability of *P. stratiotes* varied depending on the DNA methylation status of both *P. stratiotes* and *E. crassipes*. These clonal parental effects were related to resource provisioning and/or DNA methylation. We conclude that clonal parental nutrient effects can regulate interspecific competition between *P. stratiotes* and *E. crassipes* by altering the competitive ability of *P. stratiotes*. Both resource provisioning and epigenetic mechanisms can be involved in these clonal parental effects. By regulating interspecific competition, clonal parental effects may further influence species coexistence, community structure, and ecosystem functioning.

## Introduction

The environmental condition of a parent can influence the phenotype of their offspring (Wulff and Roach, 1987; Badyaev and Uller, 2009). Such a parental (environmental) effect can be transmitted to offspring generations *via* sexual propagules such as seeds (sexual parental effects) or clonal propagules such as offspring ramets (clonal parental effects) (Miao et al., 1991a; Agrawal, 2002; Dong et al., 2017; González et al., 2018; Baker et al., 2019; Luo et al., 2022). A large body of evidence shows that parental effects can influence fitness measures (e.g., growth and production) of offspring (Galloway, 2005; Latzel et al., 2014; Dong et al., 2018a,b), which may cascade to impact population- and community-level patterns and processes (Miao et al., 1991b; Bossdorf et al., 2009; Castro et al., 2013; Baker et al., 2019; Luo et al., 2022).

In nature, most plants do not grow alone, and interactions with heterospecific neighbors (i.e., interspecific interactions) are common (Grace and Tilman, 1990; Du et al., 2004). The significant role of parental effects in regulating fitness measures of offspring may further affect their interspecific interactions with heterospecific neighbors (Miao et al., 1991b; Bossdorf et al., 2009), thereby generating profound impacts on population dynamics, species coexistence, biodiversity maintenance, and ecosystem functions and services (Zou and Xu, 1998; Kraft et al., 2015; Valladares et al., 2015). If, for instance, parental effects can improve the growth of offspring (Latzel et al., 2014; Dong et al., 2017, 2018a,2019b), then they may enhance their competitive ability (Miao et al., 1991a; Bossdorf et al., 2009). On the contrary, if parental effects can reduce the growth of offspring, e.g., in some cases due to phenotypic changes (Galloway, 2005), then they may weaken their competitive ability (Bossdorf et al., 2009). However, studies on the roles of parental effects in regulating interspecific interactions are still limited (Bossdorf et al., 2009), and the few existing studies in this field focused mostly on the role of sexual parental effects in non-clonal plants (Miao et al., 1991a,b; Bossdorf et al., 2009). No studies have considered clonal parental effects on interspecific interactions (Luo et al., 2022).

Compared with non-clonal plants, clonal plants can avoid genetic variation due to meiosis, so that the epigenetic information of environmental interactions experienced by parents can be transmitted to offspring more effectively (Latzel and Klimešová, 2010; Verhoeven and Preite, 2014; Luo et al., 2022). As clonal propagules (e.g., ramets) are larger in size and mass than sexual propagules (e.g., seeds), the potential for resource provisioning might be relatively high in clonal than in non-clonal plants (Dong et al., 2019a). Therefore, parental effects may be very important for clonal plants (Verhoeven and Preite, 2014; Dodd and Douhovnikoff, 2016), particularly for those with a low ability of sexual reproduction. Thus, testing clonal parental effects on interspecific competition can deepen our understanding of the community-level roles of parental effects in clonal plants (Luo et al., 2022).

Epigenetic inheritance is an important mechanism underlying clonal parental effects (Jablonka and Raz, 2009; Latzel and Klimešová, 2010; Richards et al., 2017; González et al., 2018), and DNA methylation is one of the most important epigenetic modifications (Schulz et al., 2014). Even if the environment of offspring is different from their mother’s and the original stimulus of DNA methylation disappears, the offspring can still retain the previous methylation imprint after DNA replication (Rico et al., 2014). This epigenetic mechanism enables plants to remember past environmental experience, predict and overcome future environmental stress, and carry adaptive information through reliable transmission and selection over multiple generations (Schulz et al., 2014). By applying DNA demethylation reagent (e.g., 5-azacytidine) to plants, it was found that DNA methylation plays an important role in clonal parental effects on the morphology and growth of offspring (González et al., 2016, 2017; Portela et al., 2020). Consequently, DNA methylation may also regulate clonal parental effects on interspecific interactions. However, the epigenetic mechanism of parental effects on interspecific interactions has not been confirmed.

We conducted two consecutive experiments with two clonal floating plants (*Pistia stratiotes* and *Eichhornia crassipes*) to explore how clonal parental effects regulate interspecific interactions and the role of DNA methylation. We chose these two species because they frequently co-occur and compete with each other, and also because they can spread quickly by clonal growth. In the first experiment, the mother ramet of *P. stratiotes* and *E. crassipes* were grown in two nutrient levels and treated with a DNA demethylation reagent (5-azacytidine) or not. In the second experiment, the offspring ramets from each of the four treatments in the first experiment were grown alone (no competition) or with a heterospecific neighbor (with interspecific competition). Specifically, we tested two hypotheses: (1) clonal parental effects can alter interspecific interactions by influencing DNA methylation level; (2) the offspring produced by a mother ramet under the high nutrient condition were more competitive than those produced by a mother under the low nutrient condition, because parents under the high nutrient condition can produce offspring of higher quality.

## Materials and methods

### Study species

*Pistia stratiotes* L. (water lettuce, Araceae) is a stoloniferous floating rosette herb (Pettet and Pettet, 1970; Adomako et al., 2020, 2021). The main (i.e., vertical) stem is short with highly compressed internodes so that leaves are clustered (Odjegba and Fasidi, 2004; Adomako et al., 2021, 2022). Stolons come out from leaf axils, and new ramets are produced from stolon tips (Odjegba and Fasidi, 2004). This species is native to South America, and are now widely distributed in tropical and subtropical regions around the world, including China (Evans, 2013; Hussner et al., 2014). As one of the wetland weeds, a massive accumulation of *P. stratiotes* leads to the decline of biodiversity in local ecosystems; therefore, it has been included in the Global Invasive Species Database, and are also listed as one of the most dangerous invasive species in China (Wang et al., 2014; Galal et al., 2019).

*Eichhornia crassipes* (Mart.) Solms (water hyacinth, Pontederiaceae) is a stoloniferous floating rosette herb with a similar morphology and distribution of *P. stratiotes* (Wang et al., 2016). This species is also listed as an aggressive invasive species in many counties, including China (Wang et al., 2016), because it can quickly spread by clonal growth to form a dense mat on water surface and rapidly displace local species (Zhao, 2006). *Eichhornia crassipes* and *P. stratiotes* share similar niches and occur in lakes, rivers, ponds, and diches (Wang et al., 2016).

### Sampling and cultivation

On July 5, 2020, a clone of ramets of both *P. stratiotes* and *E. crassipes* were collected from Yongning River (28°40′3″N, 121°23′4″E) in Taizhou, Zhejiang Province, China. They were brought to a greenhouse at Taizhou University, where they were vegetatively propagated in tanks (95 cm in diameter × 60 cm in height) filled with water. On July 26, 2020, 74 newly produced offspring ramets with similar size were selected for both *P. stratiotes* and *E. crassipes* (148 ramets in total), and the stolons attached to these ramets, if any, were removed. For each species, ten of the 74 ramets were randomly selected and dried for 48 h at 70°C and weighed to measure the initial dry mass (1.08 ± 0.04 g for *P. stratiotes* and 1.32 ± 0.03 g for *E. crassipes*; mean ± SE). The remaining 64 ramets of both *P. stratiotes* and *E. crassipes* were used in the experiments described below.

### Experiment design

The study consisted of two consecutive experiments. The first experiment was launched on July 26, 2020. We randomly subjected the 64 ramets (thereafter referred to as the mother ramets) of both *P. stratiotes* and *E. crassipes* to two nutrient levels (high and low) and two DNA demethylation treatments (ramets were treated with a DNA demethylation agent or not). Thus, for both species, each of the four treatments was replicated 16 times (with 16 mother ramets). Each mother ramet was grown in a bucket (65 cm in diameter × 44 cm in height) filled with 13 L of either a high or a low nutrient solution (30 and 6% Hoagland solution, respectively). The Hoagland solution contained 945 mg/L Ca (NO_3_)_2_⋅4H_2_O, 506 mg/L KNO_3_, 80 mg/L NH_4_NO_3_, 136 mg/L KH_2_PO_4_, 493 mg/L MgSO_4_, 13.9 mg/L FeSO_4_⋅7H_2_O, and 18.7 mg/L EDTA⋅2Na. We chose these two nutrient levels because the nitrogen and phosphorous concentrations are within the gradient of nutrient levels found in water body in China (Zhang et al., 2017) and also because their difference is likely to induce a significant effect on plant growth. For DNA demethylation, 10 ml of 50 μM 5-azacitidine (5-azaC) solution was sprayed to each plant once every 3 days. For the treatment without DNA demethylation, 10 ml distilled water was sprayed.

To supplement for evapotranspiration and nutrient loss due to plant uptake, 1 L of 30% or 6% Hoagland nutrient solution was added weekly to each bucket. The first experiment lasted for 3 weeks and ended on August 16, 2020. At the end of the experiment, 2–3 offspring ramets in each bucket (produced by each mother ramet) were selected as the materials for the second experiment, and the remaining parts in each bucket were harvested to measure growth traits.

The second experiment started on 16 August 2020. For each species, the offspring ramets produced by the mother ramets grown in each of the four conditions (i.e., high and low nutrients with and without DNA demethylation) in the first experiment were randomly assigned to one of five treatments: the target ramet was grown alone (no competition, one treatment), with a ramet of a different species whose mother ramet was grown in the same condition (interspecific competition from ramets with the same parental effects; one treatment) and with a ramet of a different species whose mother ramet was grown in a different condition (interspecific competition from ramets with different parental effects; three treatments). The 16 treatments with interspecific competition were shared by the two species, resulting in a total of 24 treatments (eight treatments without competition and 16 treatments with competition).

Ramets were grown in buckets (65 cm in diameter × 44 cm in height) filled with 30% Hoagland nutrient solution. Each treatment was replicated seven times, making a total of 168 buckets with a total of 280 ramets. To compensate for water loss and nutrient consumption, 1 L of 30% Hoagland nutrient solution was added weekly to each bucket. The second experiment lasted for 3 weeks and ended on September 6, 2020. The mean temperature was 29.2°C and the mean relative humidity was 79.8% (measured hourly with a Hygrochron temperature loggers; iButton DS1923; Maxim Integrated Products, United States). At noon, the photosynthetic photon flux density at water surface was 632–1806 μmol m^–2^ s^–1^ (Li-250A; LI–COR Biosciences, United States).

### Harvest and measurements

At the end of the first experiment, we counted, for each species, the number of offspring ramets in each bucket, and measured biomass after oven-drying them at 70°C for 72 h. At the end of the second experiment, we measured biomass of each species in each bucket after drying them at 70°C for 72 h. Biomass per ramet was calculated as total biomass/number of ramets.

### Data analysis

Two-way ANOVA was used to test the effects of the nutrient level (high and low) and DNA demethylation (treated with 5-azaC or not) on total biomass, number of ramets and biomass per ramet of *P. stratiotes* and *E. crassipes* separately for the first experiment. To analyze the data from the second experiment, we first quantified the competitive response of a target plant by calculating log response ratio of biomass (LnRR), i.e., LnRR = ln(total biomass of a target ramet grown with a competing ramet/average biomass of the target ramet grown alone across the seven replicates) (Goldberg et al., 1999; Hedges et al., 1999). This calculation was carried out for each type of ramets of each species. One-sample *t*-test was used to analyze whether the mean value of the competitive response of each treatment was significantly different from zero. Then we used four-way ANOVA to examine the effects of the nutrient level and DNA demethylation of the target’s mother ramet and the nutrient level and DNA demethylation of the competitor’s mother ramet on the competition response (LnRR) of the target plant.

Before analysis, data on biomass, number of ramet and biomass per ramet were checked for normality (by Kolmogorov–Smirnov test) and homogeneity of variance (by Levene’s test). During the first experiment, one replicate of *P. stratiotes* in the low nutrient level and not treated with 5-azaC, one replicate of *E. crassipes* in high nutrient level and treated with 5-azaC and one replicate of *E. crassipes* in low nutrient level and treated with 5-azaC were completely destroyed by herbivores. During the second experiment, plants in two buckets (one with a ramet of *P. stratiotes* whose mother was grown in the high nutrient level and treated with 5-azaC and a ramet of *E. crassipes* whose mother was grown in the low level and not treated with 5-azaC, and one with a ramet of *P. stratiotes* whose mother was grown in the high nutrients level and not treated with 5-azaC and a ramet of *E. crassipes* whose mother was grown in the low nutrient level and treated with 5-azaC) were also dead. Statistical analyses were carried out with SPSS 22.0 (IBM Corp., Armonk, NY, United States).

## Results

### Effects of nutrients and DNA demethylation on performance in the first experiment

**FIGURE 1:**
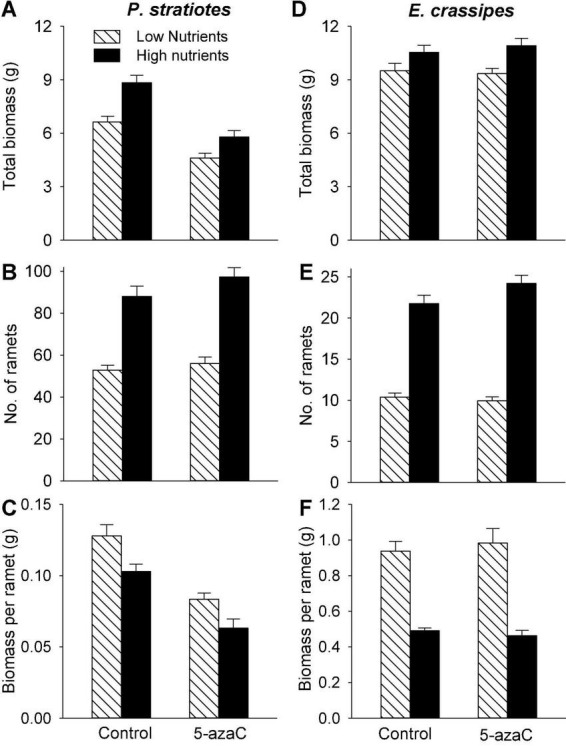
Effects of nutrient level (high and low) and DNA demethylation (control and 5-azaC) on the growth of offspring ramets of **(A–C)** *Pistia stratiotes* and **(D–F)** *Eichhornia crassipes*. Bars show means + SE.

Compared with the low nutrient level, the high nutrient level significantly increased both biomass (by 30 and 14%) and number of ramets (by 70 and 126%) of *P. stratiotes* and *E. crassipes* (Table 1 and Figures 1A,B,D,E). However, the final mean size of the ramets, as measured by biomass per ramet, was significantly higher in the low than in the high nutrient level for both species (Table 1): biomass per ramet of *P. stratiotes* was about 1.3 times larger under the low than under the high nutrient level (Figure 1C), and biomass per ramet of *E. crassipes* was about 2.0 times larger (Figure 1F). Compared to the control (no 5-azaC), the application of 5-azaC significantly reduced total biomass (by 32%) and biomass per ramet (by 37%) of *P. stratiotes*, but had no effect on ramet production (Table 1A and Figures 1A–C). The application of 5-azaC did not significantly affect total biomass, number of ramets and biomass per ramet of *E. crassipes* (Table 1B and Figures 1D–F).

**TABLE 1:** ANOVA results for effects of nutrient level and DNA demethylation on the growth of (A) *Pistia stratiotes* and (B) *Eichhornia crassipes* in the first experiment.

	Total biomass		No. of ramets		Biomass per ramet	
			
Effect	*F*	*P*	*F*	*P*	*F*	*P*
**(A) *P. stratiotes***						
Nutrient level (N)	**23.75**	**<0.001**	**96.48**	**<0.001**	**14.00**	**<0.001**
Demethylation (D)	**53.31**	**<0.001**	2.56	0.115	**48.62**	**<0.001**
N × D	2.14	0.149	0.63	0.43	0.15	0.702
**(B) *E. crassipes***						
Nutrient level (N)	**11.54**	**<0.001**	**259.85**	**<0.001**	**89.44**	**<0.001**
Demethylation (D)	0.073	0.789	1.59	0.212	0.03	0.867
N × D	0.485	0.489	3.305	0.074	0.52	0.473

Degree of freedom is 1, 59 for all effects of P. stratiotes and 1, 58 for all effects of E. crassipes. Values are in bold when P < 0.05.

### Parental effects on interspecific interactions in the second experiment

**FIGURE 2:**
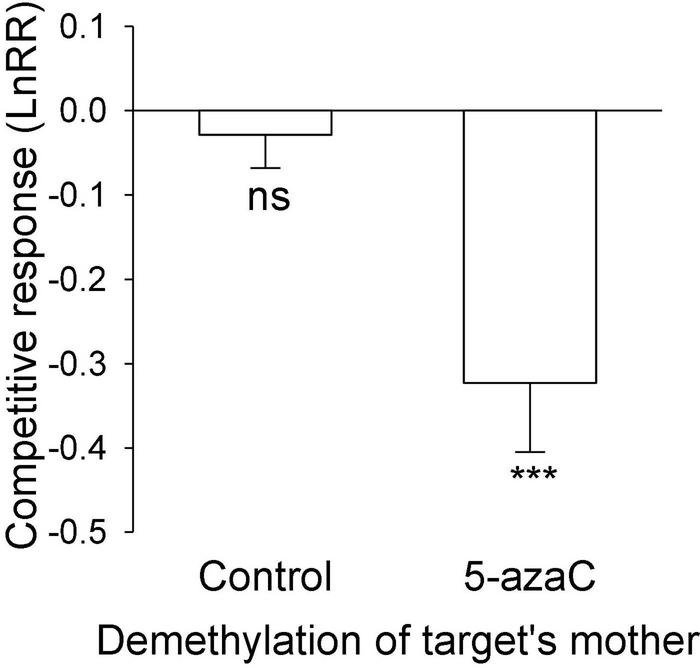
The effect of DNA demethylation of the target’s mother on the competitive response of the target plant of *Eichhornia crassipes.* Bars show means + SE. Symbols (***P < 0.001) indicate significant difference from zero (by one-sample *t*-test).

**FIGURE 3:**
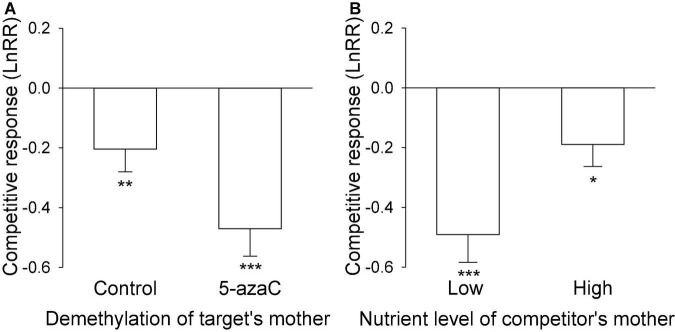
**(A)** The effect of DNA demethylation of the target’s mother and **(B)** the effect of the nutrient level of the competitor’s mother on the competitive response of the target plant of *Pistia stratiotes*. Bars show means + SE. Symbols (***P < 0.001; **P < 0.01; *P < 0.05) indicate significant difference from zero (by one-sample *t*-test).

**FIGURE 4:**
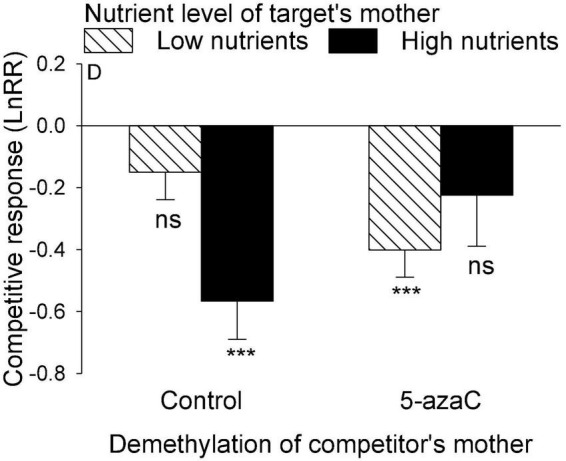
The interactive effect between DNA demethylation of the competitor’s mother and the nutrient level of the target’s mother on the competitive response of the target plant of *Pistia stratiotes*. Bars show means + SE. Symbols (***P < 0.001) indicate significant difference from zero (by one-sample *t*-test).

**FIGURE 5:**
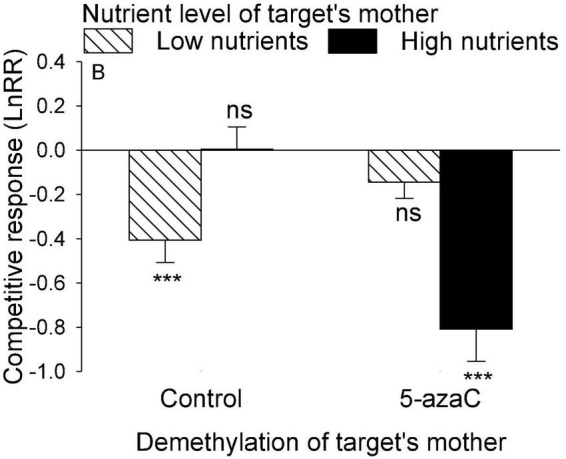
The interactive effect between nutrient level and DNA demethylation of the target’s mother on the competitive response of the target plant of *Pistia stratiotes*. Bars show means + SE. Symbols (***P < 0.001) indicate significant difference from zero (by one-sample *t*-test).

The nutrient level of the target’s mother ramet, the nutrient level of competitor’s mother ramet and the demethylation of the competitor’s mother ramet had no significant effect on the competitive response of the target plant of *P. stratiotes* (Table 2B; Appendix Figure 1). For both *E. crassipes* and *P. stratiotes*, the application of the 5-azaC to the mother ramet of the target plant significantly decreased the competitive response of the target (Table 2 and Figures 2, 3A). The competitive response of *P. stratiotes* became less negative when the competitor’s mother had been grown under the high nutrient level than when it had been grown under the low nutrient level (Table 2A and Figure 3B). We observed a significant interactive effect between the nutrient level of the target’s mother ramet and DNA demethylation of the competitor’s mother ramet and between the nutrient level of the target’s mother ramet and DNA demethylation of the target’s mother ramet on the competitive response of the target plant of *P. stratiote* (Table 2A). When the competitor’ mother was not treated with 5-azaC, the competitive response of the target plant of *P. stratiote* was not significantly different from zero if the target’ mother had been grown under the low nutrient level, but was highly significantly negative when it had been grown under the high nutrient level; however, when the target’s mother was treated with 5-azaC, a reverse pattern was observed (Figure 4). When the target’ mother was not treated with 5-azaC, the competitive response of the target plant of *P. stratiote* was significantly negative if the target’ mother had been grown under the low nutrient level, but was close to zero when it had been grown under the high nutrient level; however, when the target’s mother was treated with 5-azaC, a reverse pattern was observed (Figure 5).

**TABLE 2:** Effects of nutrient level and DNA demethylation of the target‘s mother and the competitor’s mother on the interspecific competitive response (LnRR) of the target plant of (A) *Pistia stratiotes* and (B) *Eichhornia crassipes* in the second experiment.

	(A) *P. stratiotes*		(B) *E. crassipes*	
		
Effect	*F*	*P*	*F*	*P*
Nutrient level of target’s mother (TN)	1.72	0.193	0.28	0.596
Demethylation of target’s mother (TD)	**7.59**	**0.007**	**9.98**	**0.002**
Nutrient level of competitor’s mother (CN)	**8.99**	**0.003**	0.26	0.612
Demethylation of competitor’s mother (CD)	0.10	0.754	1.16	0.284
TN × TD	**28.35**	**<0.001**	0.421	0.518
TN × CN	1.52	0.220	0.08	0.779
TN × CD	**7.76**	**0.006**	0.02	0.901
TD × CN	<0.01	0.975	2.12	0.149
TD × CD	0.434	0.512	1.06	0.306
CN × CD	1.33	0.252	0.06	0.812
TN × TD × CN	0.09	0.764	0.20	0.655
TN × TD × CD	0.95	0.334	0.39	0.534
TN × CN × CD	2.40	0.125	0.141	0.708
TD × CN × CD	0.01	0.909	0.10	0.758
TN × TD × CN × CD	1.06	0.305	1.47	0.228

Degree of freedom is 1, 94 for all effects and for both species. Values are in bold when P < 0.05.

## Discussion

### Effects of nutrient availability and 5-azaC on offspring growth

As expected, high nutrient availability of the mother ramets promoted total biomass and number of offspring ramets of both *P. stratiotes* and *E. crassipes* (Figure 1), which is consistent with previous findings on other aquatic plants (Zhao, 2006; Jampeetong and Brix, 2008; Zhang et al., 2020). This effect is related to physiological integration as mother ramets growing under higher nutrient availability can transfer more resources (photo-assimilates and nutrients) to newly produced offspring ramets that remain connected to them (Slade and Hutchings, 1987a,b; Wang et al., 2017, 2021; Portela et al., 2021). However, the magnitude of this growth promotion was stronger for number of ramets than for total biomass so that biomass per offspring ramet was significantly smaller under high than under low nutrient availability (Figures 1C,F). This result suggests that the favorable nutrient environment experienced by the mother ramets can increase the fitness of the whole offspring population, but may not necessarily promote the competitive ability of individual offspring in their subsequent growth and development.

We observed that application DNA demethylation agent 5-azaC decreased biomass per offspring ramet of *P. stratiotes* as it decreased total biomass of all offspring ramets but had no effect on their number. This finding indicates that, in addition to DNA demethylation, 5-azaC was toxic to the growth of *P. stratiotes*. A detrimental effect of 5-azaC on plant growth and development has also been reported in other species (Fieldes, 1994; Finnegan et al., 1996; Bossdorf et al., 2010), which should be considered when parental effects are subsequently considered. However, 5-azaC application had no effect on the growth of offspring ramets of *E. crassipes*, suggesting no side effect of 5-azaC on the growth of *E. crassipes*.

### Parental nutrient effects on interspecific interactions

Parental effects were found to frequently influence fitness measures of offspring (Latzel and Klimešová, 2010; González et al., 2016; Dong et al., 2019a), and thus may further influence their competitive ability when they grow with heterospecific neighbors (Miao et al., 1991a; Baker et al., 2019). For examples, parental effects improved the growth and thus competitive ability of *Polygonum persicaria* offspring in shade conditions (Baker et al., 2019). If two co-existing species both benefit from parental effects but the degree of the benefit is different (Li et al., 2021), or if parental effects can influence fitness measures of one species but had no impact on the other (Miao et al., 1991a), then parental effects can modify the competitive interactions of these co-occurring species. We found no clonal parental nutrient effects on the competitive ability of *E. crassipes* (Table 2B), but significant clonal parental nutrient effects on that of *P. stratiotes* (Table 2A and Figures 3B, 4, 5). Consequently, clonal parental nutrient effects significantly influenced the competitive interaction between *E. crassipes* and *P. stratiotes*. In a previous study, Miao et al. (1991a) reported signifiant sexual parental nutrient effects on the competitive ability of two *Plantago* species, showing the competitive ability of *Plantago major* depended on the period of parental nutrient pulse, while that of *Plantago rugelii* depended on maternal background nutrient levels. If plant communities are highly niche-differentiated, then parental effects may facilitate species coexistence (Li et al., 2021).

We observed significant parental nutrient effects of the competitor *E. crassipes* on the competitive ability of the target *P. stratiotes* (Figure 3B). The competitive ability of the offspring ramet of *P. stratiotes* became much lower when it competed with the offspring ramet of *E. crassipes* produced by the mother ramet growing under low than under high nutrient availability. It is well-known that the size of a plant is commonly positively related to its competitive ability (Goldberg and Fleetwood, 1987; Gaudet and Keddy, 1988). Because the mother ramet of *E. crassipes* produced much larger offspring ramets under low than under high nutrient availability (Figure 1F), the competitive ability of the offspring ramets originated from the mother ramet growing under low nutrients would show a much greater competitive ability when competing with *P. stratiotes*. Consequently, the parental low nutrient effect of *E. crassipes* greatly reduced the competitive ability of the offspring ramet of *P. stratiotes*. As the size of the offspring (biomass per offspring ramet) is closely related to their ability of resource provisioning (Dong et al., 2019a), the parental nutrient effect of *E. crassipes* on the competitive ability of *P. stratiotes* was likely due to resource provisioning, as reported before (Wulff and Roach, 1987; Herman and Sultan, 2011; Zas et al., 2013). However, this parental nutrient effect of *E. crassipes* was not affected by the application of 5-azaC, suggesting that DNA methylation played little role during this process (Griffin et al., 2016).

We also observed a parental nutrient effect of *P. stratiotes* on the competitive ability of its offspring ramet, but such an effect varied depending on the DNA methylation status of the mother ramet of the competitor *E. crassipes* (Figure 4). Without application of 5-azaC to the mother ramet of *E. crassipes*, the competitive ability of the offspring ramet of *P. stratiotes* was much smaller when its mother ramet had been grown under the high than under the low nutrient level. This parental nutrient effect can also be explained by resource provisioning (Wulff and Roach, 1987; Herman and Sultan, 2011; Germain et al., 2013; Zas et al., 2013) as the size of the offspring ramet of *P. stratiotes* was significantly smaller when its mother ramet had been grown under the high than under the low nutrient level (Figure 1C). However, with application of 5-azaC to the mother ramet of *E. crassipes*, the nutrient level of the mother ramet of *P. stratiotes* had no significant effect on the competitive ability of its offspring ramet, suggesting that DNA demethylation of the competitor’s mother can alter the parental nutrient effect of the target plant. The underlying mechanism for this observation is not clear and deserves further studies.

The parental nutrient effect of *P. stratiotes* on the competitive ability of its offspring ramet also varied depending on DNA demethylation status of mother ramet (Figure 5). Without application of 5-azaC to the mother ramet of *P. stratiotes*, competition from *E. crassipes* resulted in a significantly negative competitive response of the offspring ramet of *P. stratiotes* when its mother had been grown under the low nutrient level, but had no significant negative effect on the offspring ramet of *P. stratiotes* when its mother ramet had been grown under the high nutrient level. This result cannot be explained by resource provisioning (Cheplick, 1997) as the size of the offspring ramet of *P. stratiotes* was significantly smaller when its mother ramet had been grown under the high than under the low nutrient level (Figure 1C). When the mother ramet of *P. stratiotes* was treated with 5-azaC, an opposite pattern was observed (Figure 5). These results suggest that epigenetic mechanisms such as DNA methylation must have played a role in mediating the parental nutrient effect, as reported also in other studies (Latzel and Klimešová, 2010; Verhoeven and Preite, 2014; González et al., 2016).

### Parental 5-azaC effects on interspecific interactions

Average across all other treatments, application of 5-azaC to the mother ramet of *P. stratiotes* markedly decreased the competitive ability of its offspring ramets when competing with *E. crassipes* (Figure 3A), suggesting a parental effect of 5-azaC application. This parental effect was likely caused by resource provisioning as the size of the offspring ramet was significantly reduced when the mother ramet was treated with 5-azaC (Figure 1C) so that the offspring contained less energy for their subsequent growth and competition (Goldberg and Fleetwood, 1987; Gaudet and Keddy, 1988).

Surprisingly, application of 5-azaC to the mother ramet of *E. crassipes* greatly decreased the competitive ability of its offspring ramet (Figure 2), despite the fact that it did not influence the size of the offspring (Figure 1F). Thus, resource provisioning cannot explain this clonal parental effect and DNA methylation might have played a role.

## Conclusions

This appears the first study testing the role of clonal parental effects in shaping interspecific competition between plants. Our findings suggest that clonal parental nutrient effects can regulate interspecific competition between *P. stratiotes* and *E. crassipes* by altering the competitive ability of *P. stratiotes* in different ways. Both resource provisioning and epigenetic mechanisms can be involved in these clonal parental effects. By regulating interspecific competition, clonal parental effects may further influence species coexistence, community structure, and ecosystem functioning.

## Data availability statement

The raw data supporting the conclusions of this article will be made available by the authors, without undue reservation.

## Author contributions

W-HY conducted the experiment, analyzed the data, and drafted the manuscript. L-MZ assisted with data analysis and contributed substantially to manuscript revision. F-LL and M-HL contributed substantially to manuscript revision. F-HY designed the experiment and contributed substantially to manuscript revision. All authors contributed to the article and approved the submitted version.

## Conflict of interest

The authors declare that the research was conducted in the absence of any commercial or financial relationships that could be construed as a potential conflict of interest.

## Publisher’s note

All claims expressed in this article are solely those of the authors and do not necessarily represent those of their affiliated organizations, or those of the publisher, the editors and the reviewers. Any product that may be evaluated in this article, or claim that may be made by its manufacturer, is not guaranteed or endorsed by the publisher.
