# Supplementary material for: Roles of clonal parental effects in regulating interspecific competition between two floating plants
Source: Front Plant Sci. 2022 Jul 22;13:924001. doi: 10.3389/fpls.2022.924001 (PMC9355590; doi:10.3389/fpls.2022.924001)

Appendix Fig. 1 Effects of nutrient level and DNA demethylation of the target's mother and nutrient level and DNA demethylation of the competitor's mother on the interspecific competitive response (LnRR) of the target plant of (A) *Pistia stratiotes* and (B) *Eichhornia crassipes* in the second experiment. Mean and SE are given.

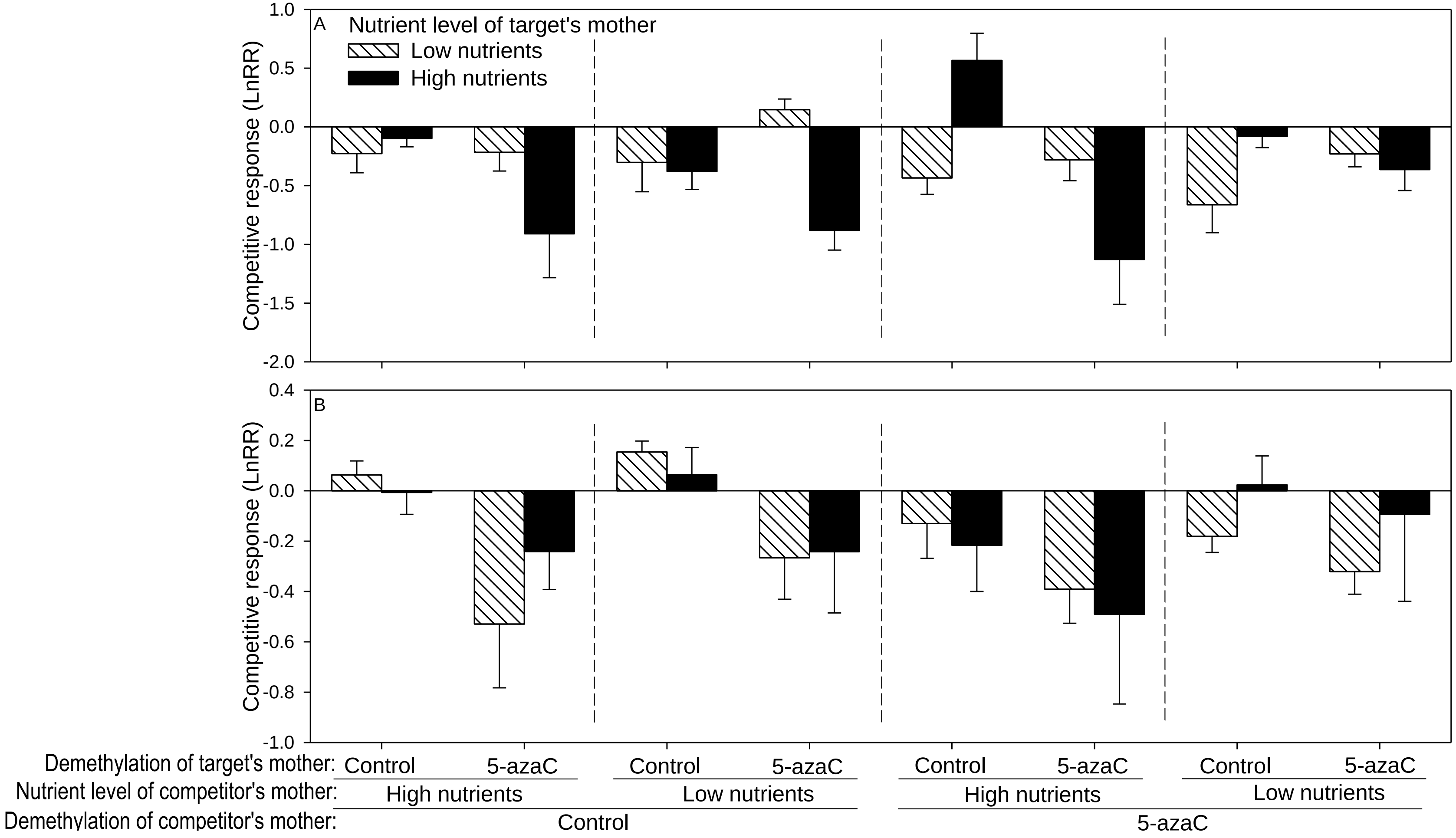

Supplement: Supplementary file 1 [file Data_Sheet_1.PDF]
